# Value of inventory information in allocating a limited supply of influenza vaccine during a pandemic

**DOI:** 10.1371/journal.pone.0206293

**Published:** 2018-10-25

**Authors:** Zihao Li, Julie L. Swann, Pinar Keskinocak

**Affiliations:** 1 School of Industrial and Systems Engineering, Georgia Institute of Technology, Atlanta, Georgia, United States of America; 2 Department of Industrial and Systems Engineering, North Carolina State University, Raleigh, North Carolina, United States of America; Columbia University, UNITED STATES

## Abstract

**Objective:**

To understand the value of information on vaccine inventory levels during an influenza pandemic, we propose a simulation study to compare vaccine allocation strategies using: (i) only population information (pro-rata, or population-based, PB), (ii) both population and vaccine inventory information (population and inventory-based, PIB).

**Methods:**

We adapt an agent-based simulation model to predict the spread of the disease both geographically and temporally. We study PB and PIB when uptake rates vary geographically. The simulation study is done from 2015 to 2017, using population and commuting data from the state of Georgia from the United States census.

**Findings:**

Compared to PB under reasonable scenarios, PIB reduces the infection attack rate from 23.4% to 22.4%, decreases the amount of leftover inventory from 827 to 152 thousand, and maintains or increases the percentage of vaccinated population.

**Conclusions:**

Our results indicate the need for greater vaccine inventory visibility in public health supply chains, especially when supply is limited, and uptake rates vary geographically. Such visibility has a potential to decrease the number of infections, help identify locations with low uptake rates and to motivate public awareness efforts.

## Introduction

Influenza in the United States has led to thousands of deaths annually over the past decade [1], and historically, there has been a world-wide pandemic about every 30 to 40 years [2]. The last declared pandemic was due to the H1N1 virus in 2009, leading to an estimated average of 363,550 deaths world-wide [3]. Timely vaccination can prevent the spread and reduce the burden of the disease.

Influenza vaccine supply is often limited, especially during a pandemic [1]; hence, vaccine allocation decisions can play a significant role in the overall impact of vaccination on reducing the disease burden. In the United States, the most recent emergency vaccine distribution campaign (2009–2010) was coordinated by the Centers for Disease Control and Prevention (CDC). As new batches of vaccine became available, they were allocated to each state proportional to the state’s population (i.e., population-based or “pro-rata” allocation) [4]. The states then distributed the vaccine inventory locally, and providers administered vaccines to individuals.

During the 2009–2010 influenza pandemic, states were encouraged to collect and report data about the vaccines administered to the general population [5]. However, very few states collected detailed information on how many vaccines were administered in each location (e.g., a county or a census tract) [6]. As a result, states did not have a good visibility into the uptake rates and vaccine inventory levels geographically. This lack of visibility in the vaccine supply chain caused some areas to end the influenza season with *excess inventory* [7, 8] (i.e., leftover vaccine) while other areas (especially those with high uptake rates) experienced *unmet demand* [9, 10].

This study aims to quantify the value of information on (or visibility into) vaccine inventory, i.e., tracking inventory levels geographically and over time, and how the use of such information in vaccine allocation decisions can improve the overall impact of vaccination. Information on the quantity of vaccines administered versus leftover in inventory in different locations could inform the decision makers on how to allocate the next batch of vaccines geographically; it could also motivate initiatives to improve access to vaccines and inform decision makers about public awareness campaigns. Such visibility could be achieved in various ways, e.g., through vaccine registries.

Combining shipment and registry information would provide visibility into administered and leftover vaccine inventory. For example, the state of Oregon collects immunization data from both public and private health care providers to create vaccination records for individuals and reports immunization rates by county for the seasonal influenza vaccine [11]. Ultimately, it is expected that the value of inventory visibility is most important when the vaccine supply is limited, which is often the case during an influenza pandemic. Vaccine inventory information, updated geographically and over time, could help reduce the disease burden by decreasing the number of infections, reduce the leftover inventory (vaccine wastage) while meeting the demand of the population in a fair and equitable manner.

## Relevant literature

Some of the literature on the allocation of limited vaccine supply focus on prioritizing certain sub-populations by age or other health risks and evaluating the benefits of targeting a limited vaccine supply [12–15], in line with the vaccine recommendations from the Advisory Committee for Immunization Practice (ACIP) in the United States, where the risk groups may be specific to an influenza strain. Some researchers have quantified the benefit from the availability and allocation of vaccines early in a pandemic [16, 17]. Prior work addressing the geographical allocation of a limited vaccine supply is scarce. Matrajt et al. propose a mathematical model to distribute vaccine in a network of cities in Southeast Asia connected by the airline transportation network; they find that a city-specific allocation strategy can reduce the attack rate substantially but at the expense of fairness [18]. Araz et al. consider the allocation of limited vaccine between and within the counties in the state of Arizona in the United States based on expected epidemic waves [19]. They find that a pro-rata strategy is effective when considering both the infection attack rate and the lead time for receiving vaccine inventory. Other authors consider shipping vaccines in two phases, where vaccines in the second phase may be sent to regions where the epidemic is not yet contained [20].

This paper proposes a modified pro-rata allocation strategy with respect to the “demand” for vaccine, by utilizing vaccine inventory information and allocating the available vaccine supply to any location where the individuals continue to request the vaccine. This is particularly important when uptake rates and vaccine coverage vary across population groups or geographically [11, 21–25]. The proposed population and inventory-based strategy thus maintains fairness with respect to the underlying demand from the population. Note that when the uptake rates are similar across geographical regions, the proposed strategy is equivalent to the traditional pro-rata (population-based) strategy; however, the proposed strategy is more effective (in terms of reducing the number of infections and leftover inventory, while maintaining fairness) when the uptake rates vary geographically.

## Methods

### Disease simulation

We adapt a simulation-based disease spread model and use data from the state of Georgia in the United States with heterogeneous population mixing to predict the spread pattern of the disease both geographically and temporally. We use a detailed Susceptible-Exposed-Infected-Recovered (SEIR) model that tracks the disease status of an individual as the disease spreads through a census-tract level contact network by interactions in households, workplaces, schools, and communities. The results presented in the main body of the paper are based on running scenarios with 10 million agents, i.e., one agent corresponding to one person in the population in the state of Georgia. (Additional results are presented in the Appendix based on running scenarios with 1 million agents, i.e., one agent corresponding to approximately 10 persons in the population.) The model is flexible and can be run with data from other locations. We consider vaccine uptake, vaccine inventory and its allocation, leftover vaccine, and total attack rate at the census tract level.

The method builds upon a previously-established agent-based simulation model [26–28]. Two main assumptions of the model are as follows (see S1 Appendix for details on how the contact network is generated):

1. Every individual is in one of following states at a given time: susceptible (*S*), exposed (*E*), pre-symptomatic (*I*_*P*_), asymptomatic (*I*_*A*_), symptomatic (*I*_*S*_), hospitalized (*H*), recovered (*R*), or dead (*D*).
2. The entire population has three levels of mixing: (i) community (day and night), (ii) peer groups (day), and (iii) household (night).

At the start of a simulation run, the entire population contact network is generated and every individual is susceptible. An initial infection is introduced randomly to 10 agents from census tracts inside Fulton County (which is in the Metropolitan Atlanta Area). An infected individual’s disease status changes to exposed (*E*). With pre-defined probabilities, the disease progresses within infected individuals and spreads to previously healthy individuals across the network. Once recovered (*R*) from the disease, the individual remains in that state.

To assess the effect of vaccination under different vaccine allocation strategies, we expand this simulation model by adding the option of vaccination; within 14 days of vaccination, a person becomes immune to the disease (i.e., moves to the recovered state) with a positive probability [1].

The simulation outputs include the spatial and temporal estimates of the spread of the disease under different vaccine allocation strategies. The total infection attack rate (IAR) represents the cumulative percentage of the population who have been infected during the epidemic. The peak prevalence is the maximum percentage of the population infected at a given time.

An important parameter in the model is *R*_0_, the reproductive number, which measures the transmission potential of the virus (i.e., the expected number of secondary infections caused by a typical infection). The analysis is presented for *R*_0_ = 1.5; similar insights are obtained for *R*_0_ = 1.8 and *R*_0_ = 2.0.

### Vaccine allocation and uptake

Since the capacity for the influenza vaccine is limited during a pandemic, vaccine supply becomes available in batches over time. Vaccine allocation begins in a “vaccination start week” and continues (e.g., on a weekly basis) until all the vaccine inventory is depleted or unmet demand reaches zero. Beginning with the vaccination start week, batches of vaccine arrive at each census tract in amounts that depend on the vaccine allocation strategy and the total vaccine availability.

The uptake rates often differ from one geographical location to another [1]. At the beginning of the simulation, we randomly select a subset of individuals (according to the uptake rate) in each census tract as willing to receive the vaccine. During each week, available vaccine is administered (randomly) to the individuals in that census tract who would like to be vaccinated, have not been infected previously, and are asymptomatic.

We consider two cases, where census tracts do and do not keep track of and report the remaining vaccine inventory levels on a weekly basis. When inventory levels are known geographically, they can inform the allocation strategy. The general principle is that areas with unused inventory could potentially receive less vaccine in the next allocation period.

We consider two strategies for allocating vaccine:

1. *Population-Based* (or pro-rata) *Strategy* (PB) delivers available vaccine in each period proportional to the population size of each census tract. This is similar to the practice followed by many states during 2009–2010 [4].
2. *Population and Inventory-Based Strategy* (PIB) allocates vaccine (in proportion to the remaining unvaccinated population in each census tract) only to those census tracts that have zero inventory, i.e., those that already administered all the vaccine that was shipped earlier.

PIB is motivated by a strategy used by organizations for allocating a limited supply of resources [29, 30]. When the uptake rates are equal in all census tracts, PB and PIB are equivalent. Detailed descriptions of both strategies are presented in S2 Appendix.

Each strategy is evaluated based on several criteria including the disease spread (e.g., IAR), operational aspects (e.g., vaccines shipped, administered, or leftover), and the “service level” (vaccine administered divided by the total number of susceptible individuals willing to receive the vaccine).

### Experiments

We ran experiments simulating various scenarios with different parameters including vaccination start week (week 4 or 7), total vaccine supply (20%, 40%, 60%, or 80% of the population), vaccine distribution horizon over which the vaccine is delivered to census tracts (in 4, 8, or 12 weeks), and three different uptake rate settings:

1. *UTR*_1_: half of the census tracts have uptake rate 25% and the other half 75%.
2. *UTR*_2_: half of the census tracts have uptake rate 0% and the other half 100%.
3. *UTR*_3_: the uptake rate of each census tract is randomly chosen from a uniform distribution between 0% and 100%.

We summarize all experimental parameters and provide justifications for their choice in Table A in S3 Appendix. To account for randomness, we generate five distinct contact networks and within each network we perform five simulation runs (replications) for each uptake rate setting (see S3 Appendix for the analysis of the number of replications needed) [31]. Hence, there are a total of 5×5 = 25 simulation runs for each of the 2×2×3×4 = 48 parameter combinations.

## Results

We present results comparing the scenarios of no vaccination, and vaccine allocation under PB and PIB for *R*_0_ = 1.5 and *UTR*_1_, focusing on IAR and the percentage of leftover (i.e., allocated but not administered) vaccine inventory. Results on *UTR*_2_ and *UTR*_3_ are presented in S4 Appendix. Unless stated otherwise, the results presented in this section are the average for the instances where the vaccination start week is 4 and the vaccine distribution horizon is 8 weeks.

### No vaccination versus vaccination under the population-based strategy

Without vaccination, the peak prevalence is 3.2%, which occurs around week 10, and the IAR is 52.0% (averaged over 25 simulation runs). The prevalence in every census tract is positive (i.e., no census tract remained free of the disease), and census tracts around the city of Atlanta have a higher IAR than rural census tracts.

When vaccine is available for 40% of the population and distributed under PB, the peak prevalence (1.6%) and IAR (26.8%) are lower and the peak occurs earlier compared to the no vaccination case (see S5 Appendix for further details). Vaccination under PB has a lower peak prevalence and IAR when vaccine is distributed earlier and over a shorter time horizon.

Selected comparisons on peak prevalence and IAR under various parameters of vaccination are presented in S6 Appendix. Detailed results for all combinations of vaccination start week, vaccine distribution horizon, and total available vaccine supply are presented in Tables A-I in S4 Appendix.

### Vaccination under population-based versus population and inventory-based strategies (PB versus PIB)

**Fig 1:**
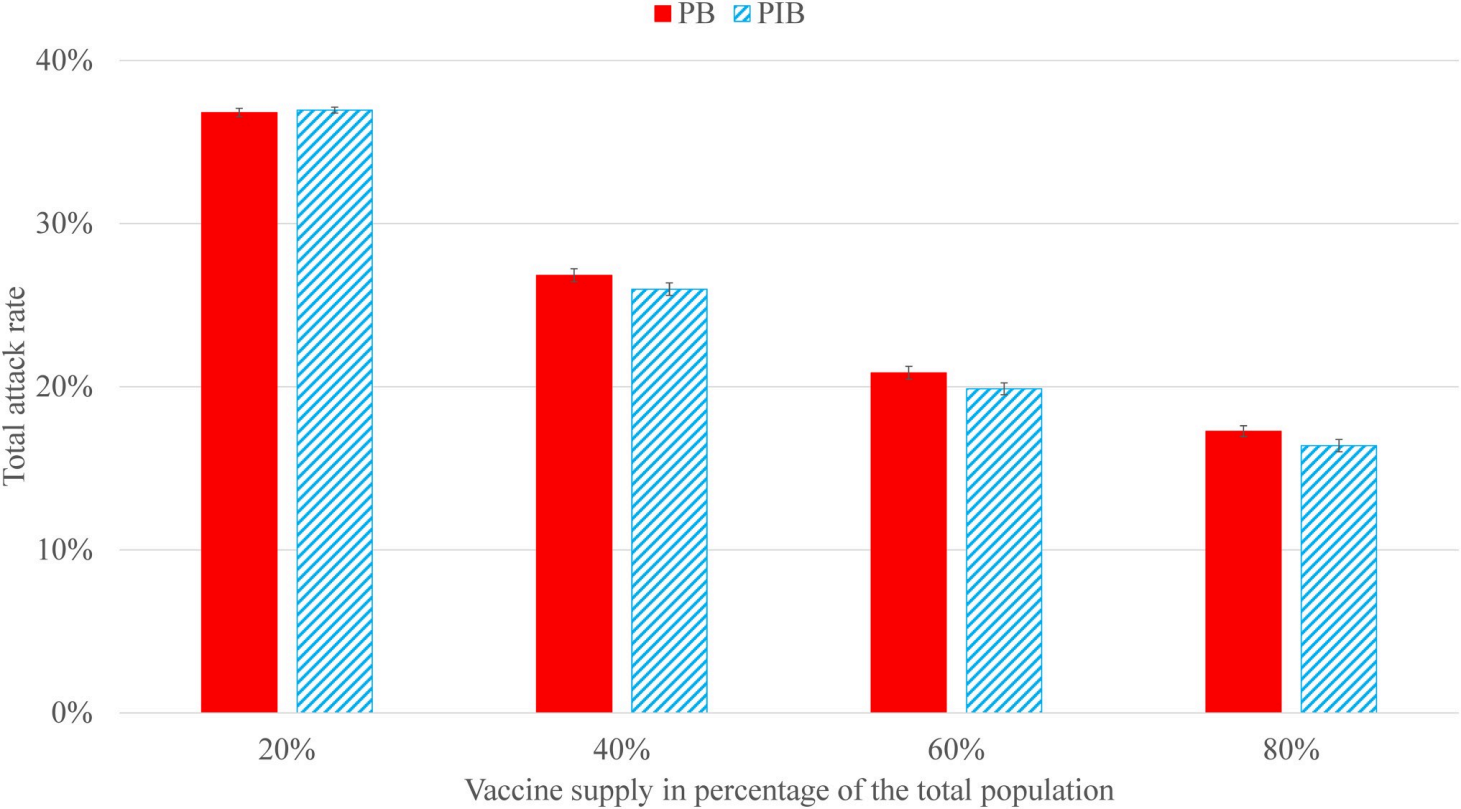
IAR under PB and PIB strategies, where the vaccination start week is 4 and the vaccine distribution horizon is 8 weeks. Error bars are the standard errors over the 50 replications for each scenario.

IAR relative to the total population under PIB is on average -0.1 to 0.9 percentage points lower than that under PB (Fig 1). These results, except for when the vaccine supply is 20% of the population, are significant under a two-sample *t*-test with a 95% confidence level. IAR under *UTR*_1_ is generally lower than that under *UTR*_2_ and *UTR*_3_ for both PB and PIB, when all other parameters are the same. The average percentage points difference in IAR between PB and PIB (PB—PIB) ranges from 4.0 to 6.4 under *UTR*_2_ and 0.9 to 1.6 under *UTR*_3_. Detailed comparisons on IAR and *t*-test results are shown in S4 Appendix.

Additional results (presented in S5 Appendix) indicate that an earlier vaccination start week and shorter vaccine distribution horizon reduce the IAR. The reductions are generally stronger for the PIB strategy.

**Fig 2:**
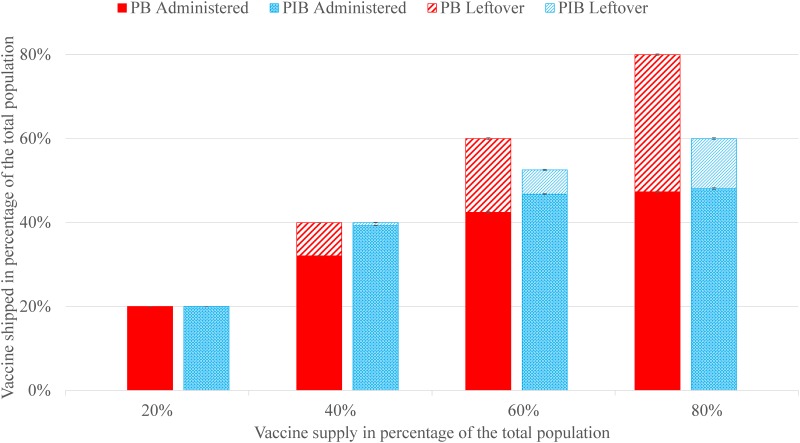
The amount of vaccine shipped, administered, and leftover in inventory, as a percentage of the total population, where the vaccination start week is 4 and the vaccine distribution horizon is 8 weeks. Error bars are the standard errors over the 50 replications for each scenario.

Fig 2 shows the number of vaccines shipped and administered under PB and PIB. The solid, striped, and combined columns represent the vaccines administered, leftover, and shipped during the entire vaccine distribution horizon, respectively.

Under PIB, the total shipment is lower or the same, but the total amount of vaccine administered is higher compared to PB; hence, vaccine utilization (and service level) is higher, and the leftover inventory is lower under PIB. For example, when the vaccine supply is equal to 40% of the population, both PB and PIB ship all the available vaccine, but PIB administers 674 thousand more doses than PB. As a result, the leftover inventory is 20.7% of the total shipped under PB versus 3.8% of the total shipped under PIB.

The cost of the leftover vaccine inventory is estimated using information from a previous study in New York City [32, 33]. The (per dose) vaccine production cost is $5.0 to $10.0, distribution cost is $1.5 to $5.0, and disposal cost is $0.1 to $1.0. When the vaccine supply is enough to cover 40% of the population, PIB ships the same amount of vaccine and results in 667 thousand (95% CI on PIB-PB, 664 to 669, *p* < 0.0001) less doses of leftover vaccine (or equivalently, more doses of administered vaccine) compared to PB (Fig 1). Hence, the estimated cost savings under PIB versus PB range from $4.4 to $10.7 million dollars.

We also calculate the service levels under PB and PIB. “*Demand*” refers to the number of people who were willing to receive the vaccine (calculated by multiplying the population in an area with the uptake rate), and “*Served*” refers to the number of people who were vaccinated. *Service level* is defined as $\frac{Served}{Demand}\times 100\%.$ When the vaccine supply is sufficient to cover 40% of the population, the mean service levels under PB and PIB for census tracts with 75% uptake rate are 58.6% and 75.9% (95% CI of the percentage points difference on PIB-PB, 13.3 to 21.3, *p* < 0.0001), respectively. For census tracts with 25% uptake rate, the average service level for PB and PIB are 93.2% and 95.0% (-2.1 to 5.8, *p* = 0.3411), respectively. Additional results and details are presented in S6 Appendix.

## Discussion

The results indicate that PIB dominates PB across multiple metrics. Given a fixed amount of vaccine supply, more vaccine is administered to the population (higher service levels), resulting in similar or lower IAR under PIB versus PB. Since PIB ships the vaccine (over time, as new batches become available) to those areas where there is still demand for the vaccine, versus shipping it to areas where the demand is saturated, it ships less vaccine, and hence, incurs a lower transportation cost and lower amount of leftover inventory, compared to PB. Note that the percentage of population vaccinated in any geographic area is higher under PIB versus PB; hence, the benefits of PIB are realized while maintaining fairness.

IAR under PIB is similar to or lower than that under PB. A 0.8% drop in IAR (with vaccine supply of 40% in Fig 2) implies approximately 100 thousand fewer influenza cases in the state of Georgia. In general, the reduction in IAR under PIB versus PB positively correlates with the variability in the uptake rates across locations (IAR reduction is the highest under *UTR*_2_ and the lowest under *UTR*_1_), i.e., the higher the variability in the uptake rates, the higher the benefits of PIB over PB. Additional results and discussions on the changes in IAR when the uptake rates are correlated geographically or when considering herd immunity can be found in S6 Appendix.

Leftover (unused, and potentially wasted) vaccine inventory often incurs extra cost (including storage and disposal), as experienced during the last phase of the H1N1 influenza vaccine campaign [34–36]. These costs are even higher if the leftover vaccine is treated as a hazardous waste, as was required in some states for vaccine containing thimerosal. Inventory visibility enables the implementation of allocation strategies such as PIB, reducing the amount and the cost of leftover vaccine inventory, and the potential negative environmental impact.

Visibility in inventory has additional benefits that have not been explored in this study. For example, inventory information can be used to learn the uptake rate in each census tract, and states could design policies or information campaigns for areas with low uptake rates to create awareness, which could result in an increase in vaccination rates across the population and greater reductions in IAR. The lower number of infections along with the cost savings enabled by PIB would free up valuable resources which could be invested elsewhere to improve the availability and access to public health services.

Overall, this study suggests that visibility of inventory information in public health supply chains can have many benefits. Some states have adopted systems and practices to increase visibility in supply chains [11], and others may want to consider the potential benefits and costs of such practices. Ultimately, investments towards increasing the visibility in public health supply chains could increase effectiveness (reducing the disease burden) and efficiency (saving cost), while promoting equity (fairness).

## Limitations

We assume that the uptake rates, i.e., the willingness to receive the vaccine, are constant over time (but we allow the uptake rates to vary by location). In practice, uptake rates may vary over time, e.g., they may be lower towards the end of the epidemic. Since we limit the vaccine distribution horizon to several weeks around the peak (where one would expect the awareness about the epidemic to be high) it is reasonable to assume that the uptake rates would be somewhat stable during the vaccine distribution horizon. Uptake rates could lag behind the actual demand of the vaccine at the beginning of the pandemic. For example, poor logistics or limited vaccine availability could slow the distribution of vaccine to individual vaccine providers, or the public may not realize the availability of the vaccine due to inadequate marketing or public outreach. If the vaccine supply is very limited at the beginning, and people try to get vaccinated but are not able to do so after repeated tries, they may give up after a while and this could also reduce uptake rates. On the other hand, the severity of the epidemic could cause uptake rates to increase over time as the public becomes more informed about the spread and the danger of the disease. The benefits of inventory visibility could be even higher when the uptake rates vary over time. For example, a decreasing trend in uptake rates might prompt a local government to increase efforts in generating public awareness regarding the benefits of vaccination, and conversely, and increasing trend could be communicated to the upper levels of the vaccine supply chain to ensure inventory availability.

The current model considers a single type of influenza vaccine. In practice, there may be multiple types of vaccines, and there may be some overlap between the populations who are eligible to receive different types of vaccines. We expect that the PIB strategy would perform better than the PB strategy, even if the uptake rates differ among various vaccine types and geographical locations. In the models, we track the vaccine inventory in a semi-aggregate fashion for computational efficiency (e.g., vaccine inventory levels are computed for about 1,600 census tracts in the model vs. more than ten thousand providers in practice). There are well-known results in the supply chain management literature indicating that as the number of locations holding the inventory increases, stocking levels and costs increase (to meet a desired service level), or alternatively, service levels decrease for a given fixed amount of inventory. Hence, in practice, given a high number of inventory locations and potentially higher variability between uptake rates among different locations, we expect that PIB could be even more beneficial compared to PB.

Although we have demonstrated that PIB can reduce the cost associated with vaccine distribution and leftover inventory, it is still necessary to estimate the cost of implementing an information system that could track vaccine inventory at a local level to assess when PIB would be cost-saving compared to PB. Such a system may be relatively easy to implement, e.g., as an add-on to vaccine registries across the states.

## Supporting information

S1 Appendix Illustration of contact network generation and disease progression. (DOCX)

S2 Appendix Detailed description of vaccine inventory allocation strategies. (DOCX)

S3 Appendix Parameters of the simulation model. (DOCX)

S4 Appendix Detailed comparison between vaccine inventory allocation strategies under multiple scenarios. (DOCX)

S5 Appendix Detailed analysis on no vaccination vs. vaccination under PB (vaccine supply is 40% population). (DOCX)

S6 Appendix Additional analysis and discussion on PB vs. PIB. (DOCX)

## References

[1] Key Facts About Seasonal Flu Vaccine: Centers for Disease Control and Prevention; 2014 [updated October 22, 2014; cited 2015 June 8]. http://www.cdc.gov/flu/protect/keyfacts.htm.

[2] Taubenberger JK, Morens DM. Influenza: the once and future pandemic. Public Health Rep. 2010;125 Suppl 3:16–26. 20568566 PMC2862331

[3] Dawood FS, Iuliano AD, Reed C, Meltzer MI, Shay DK, Cheng PY, et al Estimated global mortality associated with the first 12 months of 2009 pandemic influenza A H1N1 virus circulation: a modelling study. Lancet Infectious Diseases. 2012;12(9):687–95. 10.1016/S1473-3099(12)70121-4 22738893

[4] H1N1 Flu Allocation and Distribution Q&A: Centers for Disease Control and Intervention; 2009 [cited 2017 July 17]. http://www.cdc.gov/H1N1flu/vaccination/statelocal/centralized_distribution_qa.htm.

[5] CDC 2009 H1N1 Vaccination Campaign Planning Checklist: Centers for Disease Control and Prevention; 2009 [cited 2017 July 17]. https://www.cdc.gov/h1n1flu/vaccination/statelocal/planning_checklist.htm.

[6] Stroud C, Nadig L. Data Collection, Monitoring, Evaluation, and Use. In: Altevogt BM, editor. The 2009 H1N1 Influenza Vaccination Campaign: Summary of a Workshop Series. Washington, D.C.: THE NATIONAL ACADEMIES PRESS; 2011. 21595118

[7] Evans J. Happy Trails, Swine Flu Vaccine. 2010.

[8] Stobbe M. Expired swine flu shots amount to $260 million loss: NBCNEWS.com; 2010. http://www.nbcnews.com/id/38046491/ns/health-cold_and_flu/t/expired-swine-flu-shots-amount-million-loss/#.WvrlUoWcHmQ.

[9] Shear MD, Stein R. Administration officials blame shortage of H1N1 vaccine on manufacturers, science Washington, DC: Washington Post; 2009 [cited 2018 May 15]. http://www.washingtonpost.com/wp-dyn/content/article/2009/10/26/AR2009102603487.html.

[10] Knox R. Swine Flu Vaccine Shortage: Why?: NPR.org; 2009. https://www.npr.org/templates/story/story.php?storyId=114156775.

[11] Oregon.gov. Oregon Seasonal Flu Vaccine Uptake 2014 [cited 2016 2016/01/04]. https://public.health.oregon.gov/PreventionWellness/VaccinesImmunization/Pages/FluVaccine.aspx.

[12] Drakopoulos K, Ozdaglar A, Tsitsiklis J, Ieee. An efficient curing policy for epidemics on graphs. 2014 Ieee 53rd Annual Conference on Decision and Control (Cdc). 2014:4447–54.

[13] Mbah MLN, Medlock J, Meyers LA, Galvani AP, Townsend JP. Optimal targeting of seasonal influenza vaccination toward younger ages is robust to parameter uncertainty. Vaccine. 2013;31(30):3079–89. 10.1016/j.vaccine.2013.04.052 23684837 PMC3764605

[14] Medlock J, Galvani AP. Optimizing Influenza Vaccine Distribution. Science. 2009;325(5948):1705–8. 10.1126/science.1175570 19696313

[15] Mylius SD, Hagenaars TJ, Lugner AK, Wallinga J. Optimal allocation of pandemic influenza vaccine depends on age, risk and timing. Vaccine. 2008;26(29–30):3742–9. 10.1016/j.vaccine.2008.04.043 18524428

[16] Conway JM, Tuite AR, Fisman DN, Hupert N, Meza R, Davoudi B, et al Vaccination against 2009 pandemic H1N1 in a population dynamical model of Vancouver, Canada: timing is everything. Bmc Public Health. 2011;11 10.1186/1471-2458-11-932 22168242 PMC3280345

[17] Matrajt L, Longini IM Jr. Optimizing Vaccine Allocation at Different Points in Time during an Epidemic. Plos One. 2010;5(11). 10.1371/journal.pone.0013767 21085686 PMC2978681

[18] Matrajt L, Halloran ME, Longini IM Jr. Optimal Vaccine Allocation for the Early Mitigation of Pandemic Influenza. Plos Computational Biology. 2013;9(3). 10.1371/journal.pcbi.1002964 23555207 PMC3605056

[19] Araz OM, Galvani A, Meyers LA. Geographic prioritization of distributing pandemic influenza vaccines. Health Care Management Science. 2012;15(3):175–87. 10.1007/s10729-012-9199-6 22618029 PMC4295509

[20] Yarmand H, Ivy JS, Denton B, Lloyd AL. Optimal two-phase vaccine allocation to geographically different regions under uncertainty. European Journal of Operational Research. 2014;233(1):208–19. 10.1016/j.ejor.2013.08.027

[21] Egede LE, Zheng DY. Racial/ethnic differences in influenza vaccination coverage in high-risk adults. American Journal of Public Health. 2003;93(12):2074–8. 10.2105/ajph.93.12.2074 14652337 PMC1448155

[22] Rasmussen SA, Jamieson DJ, MacFarlane K, Cragan JD, Williams J, Henderson Z, et al Pandemic Influenza and Pregnant Women: Summary of a Meeting of Experts. American Journal of Public Health. 2009;99:S248–S54. 10.2105/AJPH.2008.152900 19461110 PMC4504360

[23] Lu PJ, Ding H, Euler GL, Furlow C, Bryan LN, Bardenheier B, et al Interim Results: State-Specific Influenza A (H1N1) 2009 Monovalent Vaccination Coverage—United States, October 2009-January 2010. Morbidity and Mortality Weekly Report. 2010;59(12):363–8. 20360670

[24] Galarce EM, Minsky S, Viswanath K. Socioeconomic status, demographics, beliefs and A(H1N1) vaccine uptake in the United States. Vaccine. 2011;29(32):5284–9. 10.1016/j.vaccine.2011.05.014 21621577

[25] Uscher-Pines L, Maurer J, Harris KM. Racial and Ethnic Disparities in Uptake and Location of Vaccination for 2009-H1N1 and Seasonal Influenza. American Journal of Public Health. 2011;101(7):1252–5. 10.2105/AJPH.2011.300133 21566026 PMC3110237

[26] Ekici A, Keskinocak P, Swann JL. Modeling Influenza Pandemic and Planning Food Distribution. M&Som-Manufacturing & Service Operations Management. 2014;16(1):11–27. 10.1287/msom.2013.0460

[27] Shi P, Keskinocak P, Swann JL, Lee BY. The impact of mass gatherings and holiday traveling on the course of an influenza pandemic: a computational model. Bmc Public Health. 2010;10 10.1186/1471-2458-10-778 21176155 PMC3022852

[28] Shi P, Keskinocak P, Swann JL, Lee BY. Modelling seasonality and viral mutation to predict the course of an influenza pandemic. Epidemiology and Infection. 2010;138(10):1472–81. 10.1017/S0950268810000300 20158932 PMC3779923

[29] Cachon GP, Lariviere MA. Capacity allocation using past sales: When to turn-and-earn. Management Science. 1999;45(5):685–703. 10.1287/mnsc.45.5.685

[30] Lawrence D. GM test allocation change: More makers scrap turn-and-earn plan: Automotive News; 1996 [cited 2016 Jan 04]. http://www.autonews.com/article/19960212/ANA/602120755/gm-tests-allocation-change:more-makers-scrap-turn-and-earn-plan.

[31] Bellan SE, Pulliam JRC, Pearson CAB, Champredon D, Fox SJ, Skrip L, et al Statistical power and validity of Ebola vaccine trials in Sierra Leone: a simulation study of trial design and analysis. Lancet Infectious Diseases. 2015;15(6):703–10. 10.1016/S1473-3099(15)70139-8 25886798 PMC4815262

[32] Glazner JE, Beaty B, Berman S. Cost of vaccine administration among pediatric practices. Pediatrics. 2009;124 Suppl 5:S492–8. 10.1542/peds.2009-1542H 19948580

[33] Kansagra SM, McGinty MD, Morgenthau BM, Marquez ML, Rosselli-Fraschilla A, Zucker JR, et al Cost Comparison of 2 Mass Vaccination Campaigns Against Influenza A H1N1 in New York City. American Journal of Public Health. 2012;102(7):1378–83. 10.2105/AJPH.2011.300363 22676501 PMC3478017

[34] Influenza A (H1N1) 2009 Monovalent Vaccine Storage, Preparation, Handling Q & A: Centers for Disease Control and Prevention; 2010 [cited 2016 Jan 4]. http://www.cdc.gov/h1n1flu/vaccination/storage_handling_qa.htm#e.

[35] Disposal of Expired/Wasted H1N1 Vaccine: Illinois Department of Public Health; 2010 [cited 2016 Jan 4]. http://www.idph.state.il.us/h1n1_flu/177-H1N1_vaccine_disposal_012510.pdf.

[36] Disposing of Unused or Expired H1N1 Vaccine in Minnesota Minnesota Department of Health; 2010 [cited 2016 Jan 4]. http://www.mnhlrp.org/images/VaccineDisposal.pdf.

