# Supplementary material for: Value of inventory information in allocating a limited supply of influenza vaccine during a pandemic
Source: PLoS One. 2018 Oct 25;13(10):e0206293. doi: 10.1371/journal.pone.0206293 (PMC6201932; doi:10.1371/journal.pone.0206293)
Supplement: S1 Appendix — (DOCX) [file pone.0206293.s001.docx]

In our agent-based model, each agent has certain attributes (e.g., age and commuting pattern) and is assigned to a specific census tract with certain attributes (e.g., age and commuting pattern). In simulations with 1 million agents, one agent represents approximately ten people in the same census tract with similar attributes. We run the simulation with roughly one million agents and count the number of agents being infected at each day. The IAR is then computed by using the number of infected agents divided by the total number of agents in that census tract. The county-level IAR is an average over all the census tracts in that county. Running one instance of the simulation with one million agents takes about 10-15 minutes, and about two hours with 10 million agents. Given the very large number of simulation runs (there are a total of 3,600 runs in our computational study), doing each run with 10 million agents would take very long time; hence, we chose to run the simulation with ten million agents for the scenarios presented in the main body of the paper, and one million agents for the scenarios presented in S1-S6 Appendix. Next, we explain that for a given scenario, the results from the simulation runs with one million agents are similar to those from the runs with 10 million agents.

To ensure the validity of the results, we run 25 replications of the scenario when the vaccination start week is 4, vaccine distribution horizon is 8 weeks, and the total supply is 40% of the population with roughly 10 million agents. For the simulation runs with 10 million agents, the average differences in percentage points of IAR, the amount of vaccines administered and leftover as a percentage of population, and service level for census tracts with 75% uptake rate between PB and PIB are 0.8 (p = 0.0000), 7.2 (p = 0.0000), 7.2 (p = 0.0000), and 17.3, respectively. For the simulation runs with one million agents under the same scenario, the differences are 1.0 (p = 0.0000), 6.7 (p = 0.0000), 6.7 (p = 0.0000), and 17.8. The p-values are all < 0.0001 in each comparison. The quantitative comparison shows that results are consistent between the 1 million agents and 10 million agents simulation runs.

The spread of the disease among the susceptible population is modeled using a contact network. We stratify the entire population by age (0-5, 6-11, 12-18, 19-64, and 65+), geography (at a census tract level), and households (size determined using Georgia census data) and assign each individual to communities that correspond to neighborhoods.

A susceptible individual can be infected by other individuals in the same household, peer group, or the same community with varying probabilities. During the day, we model interactions in peer groups or community (e.g., grocery stores, restaurants, churches, and other areas where people get together). For example, a percentage of adults (19-64) commute to work in a different community, interacting with other adults who also work in that community (we utilized data on county-to-county commuting patterns from the United States Census Bureau). Children interact with peers at schools, which is captured in the simulation by utilizing data on age groups, kindergarten (0-5), elementary (6-11), and secondary (12-18) schools. The elderly (65+) interact in the household (which could be a retirement home) or community, but do not mix in peer groups such as work or school. While peer group and community networks model day–time interactions, the model captures night-time interactions (and resulting infection transmissions) as well, by allowing individuals who live in the same household to have contact with and infect each other during night time.

We illustrate the contact network in our model in Fig A.

**
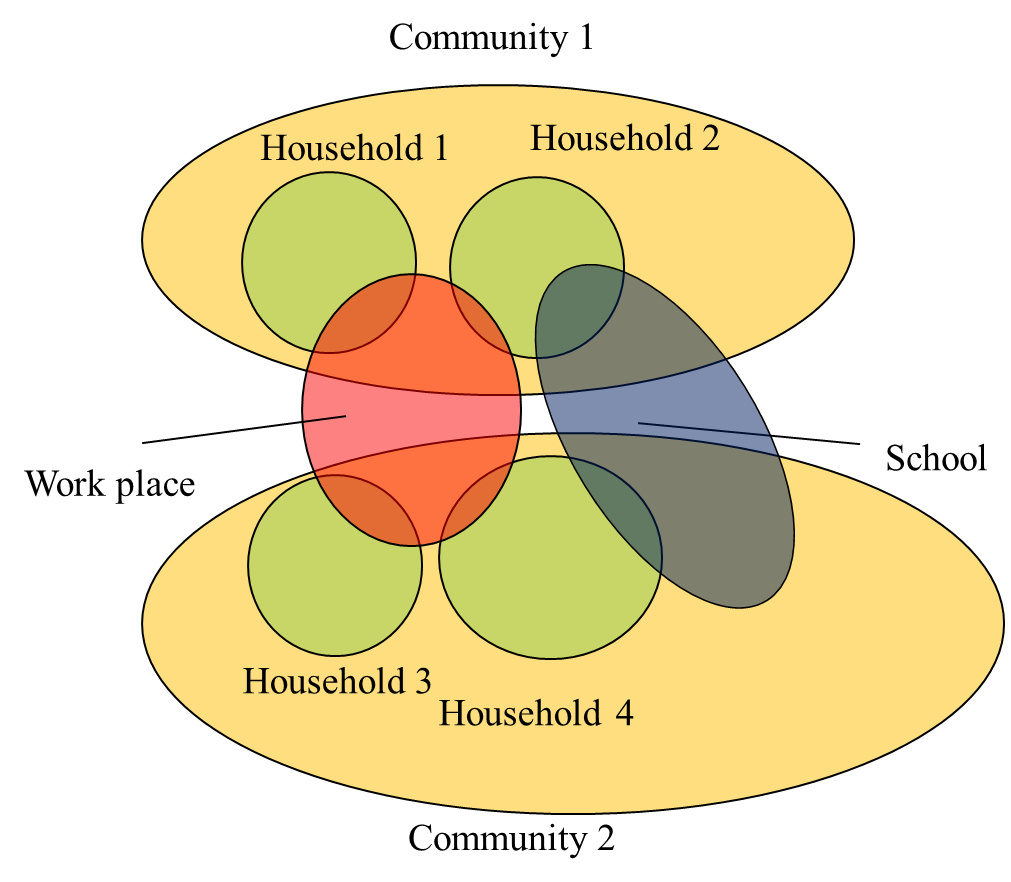
**

**Fig A: Example of a contact network.**

In Fig B we illustrate the natural progression of influenza within each individual.

*
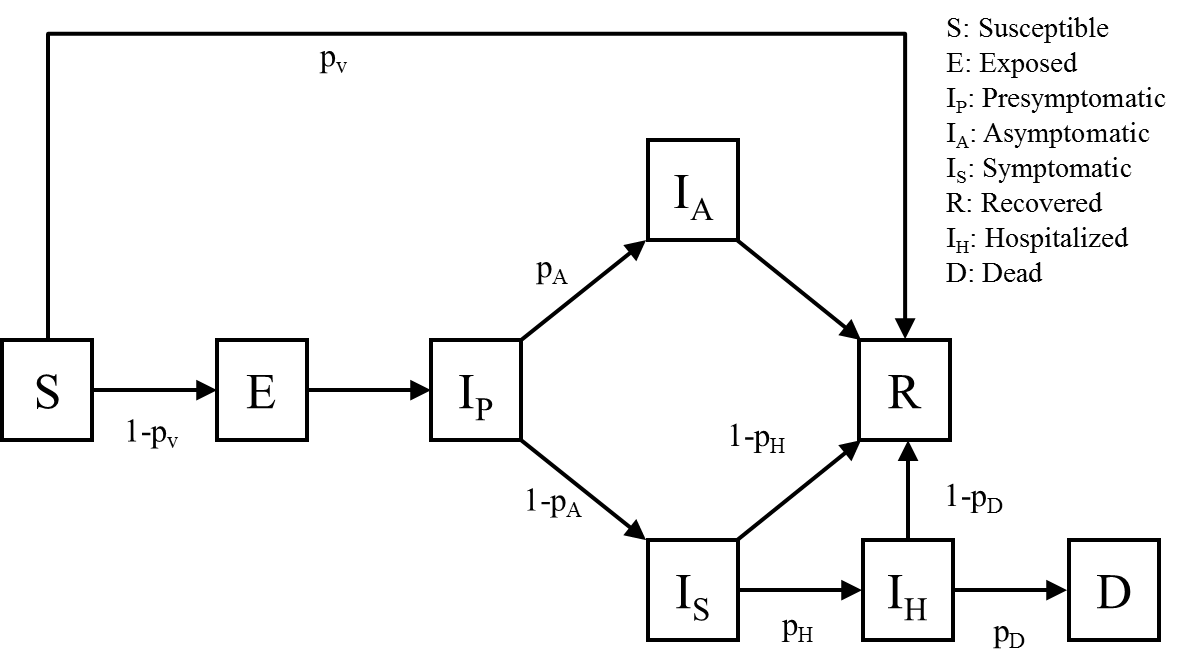
*

**Fig B: Natural progression of the disease.**

Parameters in Fig 2:

$p_{A}=$ 0.4 for adults (19-64) and 0.25 for others [[1-4](#_ENREF_1)]

$p_{H}=$ 0.18 for children between zero and five years; 0.12 for elderly (65+) and 0.06 for others [[3](#_ENREF_3), [4](#_ENREF_4)]

$p_{D}=$ 0.344 for elderly and children between zeros and five years and 0.172 for others [[4](#_ENREF_4), [5](#_ENREF_5)]

$p_{v}=$ 0.75 for individuals who have been vaccinated two weeks ago, 0 for others [[6](#_ENREF_6)]

Duration of $E+I_{P} \sim Weibull$(1.48, 0.47) (including an offset of 0$.$5 days) [[4](#_ENREF_4), [7](#_ENREF_7)]

Duration of $I_{P}=$ 0.5 days [[4](#_ENREF_4), [7](#_ENREF_7)]

Duration of $I_{S} \sim$ Exponential(2.73) [[4](#_ENREF_4)]

Duration of $I_{A} \sim$ Exponential(1.64) [[4](#_ENREF_4)]

Duration of $I_{H} \sim$ Exponential(14) [[4](#_ENREF_4), [7](#_ENREF_7)]

**References:**

1. Ferguson NM, Mallett S, Jackson H, Roberts N, Ward P. A population-dynamic model for evaluating the potential spread of drug-resistant influenza virus infections during community-based use of antivirals. Journal of Antimicrobial Chemotherapy. 2003;51(4):977-90. doi: 10.1093/jac/dkg136. PubMed PMID: WOS:000182392900026.

2. Germann TC, Kadau K, Longini IM, Macken CA. Mitigation strategies for pandemic influenza in the United States. Proceedings of the National Academy of Sciences of the United States of America. 2006;103(15):5935-40. doi: 10.1073/pnas.0601266103. PubMed PMID: WOS:000236896200054.

3. Longini IM, Nizam A, Xu SF, Ungchusak K, Hanshaoworakul W, Cummings DAT, et al. Containing pandemic influenza at the source. Science. 2005;309(5737):1083-7. doi: 10.1126/science.1115717. PubMed PMID: WOS:000231230100048.

4. Wu JT, Riley S, Fraser C, Leung GM. Reducing the impact of the next influenza pandemic using household-based public health interventions. Plos Medicine. 2006;3(9):1532-40. doi: 10.1371/journal.pmed.0030361. PubMed PMID: WOS:000241923800021.

5. Carrat F, Luong J, Lao H, Salle AV, Lajaunie C, Wackernagel H. A 'small-world-like' model for comparing interventions aimed at preventing and controlling influenza pandemics. Bmc Medicine. 2006;4. doi: 10.1186/1741-7015-4-26. PubMed PMID: WOS:000244871900001.

6. Key Facts About Seasonal Flu Vaccine: Centers for Disease Control and Prevention; 2014 [updated October 22, 2014; cited 2015 June 8]. Available from: <http://www.cdc.gov/flu/protect/keyfacts.htm>.

7. Ferguson NM, Cummings DAT, Cauchemez S, Fraser C, Riley S, Meeyai A, et al. Strategies for containing an emerging influenza pandemic in Southeast Asia. Nature. 2005;437(7056):209-14. doi: 10.1038/nature04017. PubMed PMID: WOS:000231696900046.
