# Supplementary material for: Value of inventory information in allocating a limited supply of influenza vaccine during a pandemic
Source: PLoS One. 2018 Oct 25;13(10):e0206293. doi: 10.1371/journal.pone.0206293 (PMC6201932; doi:10.1371/journal.pone.0206293)
Supplement: S2 Appendix — (DOCX) [file pone.0206293.s002.docx]

We assume that the vaccine supply is available at the beginning of a given week $w$ and the total amount of vaccine available is $T_{w}$. For each census tract $i$ in week $w$, we have the population $p_{i}$, vaccine inventory level $v_{i}^{w}(v_{i}^{1}=0,\forall i)$ and the total amount of vaccine shipped prior to week $t_{i}^{w}$. We calculate the potential size of the population that are eligible for vaccination as follows:

$s_{i}^{w}=p_{i}-\sum_{k<w} t_{i}^{k}$.

Let $r_{i}^{w}$ denote the vaccine “requirement” in census tract $i$ in week $w$.

Under PB: $r_{i}^{w}=p_{i},$ for each census tract $i$ and each week $w$.

Under PIB: $r_{i}^{1}=p_{i}$ for each census tract $i$ and for $w\geq$ 2, $r_{i}^{w}=\left\{ \begin{aligned} t_{i}^{w}, &v_{i}^{w}\leq0 \\ 0, &v_{i}^{w}>0 \end{aligned} \right.$.

Note that the requirement under PIB is in line with the “demand,” i.e., the number of people who are willing to receive the vaccine, who have not been vaccinated, and who have not been infected and recovered. Both policies allocate $a_{i}^{w}=\min\left\{ \frac{r_{i}^{w}}{\sum_{i} r_{i}^{w}},s_{i}^{w} \right\}$ amount of vaccine to each census tract $i$. In both strategies, fractional levels of vaccine are rounded down to the nearest integer and if there is any vaccine left, they are distributed proportionally to census tracts that still demand vaccine.
