# Supplementary material for: Value of inventory information in allocating a limited supply of influenza vaccine during a pandemic
Source: PLoS One. 2018 Oct 25;13(10):e0206293. doi: 10.1371/journal.pone.0206293 (PMC6201932; doi:10.1371/journal.pone.0206293)
Supplement: S3 Appendix — (DOCX) [file pone.0206293.s003.docx]

During the 2009-2010 influenza pandemic, the vaccine supply (~120 million doses) was sufficient to cover about 40% of the population (~300 million) in the United States. Vaccine was delivered starting around week 40 of 2009 [[1](#_ENREF_1)]. For the seasonal influenza vaccine, the uptake rate during the 2009-2010 season was 40.4% for adults, and 43.4% for children [[2](#_ENREF_2)]. We used this information to parameterize the simulation model.

**Table A: Parameters for vaccination.**

| Start Week | 4 and 7 | Vaccine distribution horizon | 4, 8, and 12 weeks. |
| --- | --- | --- | --- |
| Total Supply | 20%, 40%, 60%, and 80% of total population | Vaccine Effectiveness | 75% [[3](#_ENREF_3)] |
| Uptake Rate | ½ with 0, ½ with 1,  ½ with 25%, ½ with 75%,  Uniformly distributed from 0 to 1 | Show up rate | 100% |
| $R_{0}$ | 1.5, 1.8, 2.0 |  |  |

We ran simulations with full combinations of each parameter in Table A and 1 million agents for results presented in S1-S6 Appendix. The number of replications for each combination is $N=$50. Although the same 10 uptake rate instances are used for all the 5 networks, the 50 IARs pass all normality test and there are no correlations between networks with the same uptake rate instance. The maximum sampled standard deviation for all scenarios is $\sigma=$ 0.015. On a confidence interval of 95%, which corresponds to a$z$-value of 1.96, the width is $z\cdot\frac{\sigma}{\sqrt{N}} =$0$.$004 or 0.4% of total population. For results presented in the main document, we ran 25 replications for each combination of parameter in Table 1 under $UTR_{1}$ with 10 million agents.

The solid line in Fig A shows the percentage of total hospital visits that are due to influenza like illnesses (ILI) during the 2009-2010 influenza pandemic and the two double lines indicate the time periods for vaccination in our simulation when the vaccination start week is 4 or 7 and vaccines are distributed for 8 weeks. As a comparison, the dashed straight line is the national baseline for the percentage of hospital visits that are due to ILI during the regular influenza season.

Week 12

Week 15

Week 7

Week 4

**Fig A: Percentage of visits for influenza like illness 2009 to 2010 season [**[**4**](#_ENREF_4)**]. Week one corresponds to the 35^th^ week of 2009.**

**References**

1. 2009 H1N1 Influenza Vaccine Supply Status: Centers for Disease Control and Prevention; 2009 [cited 2017 Mar 24]. Available from: <https://www.cdc.gov/h1n1flu/vaccination/updates/101609.htm>.

2. Flu Vaccination Coverage, United States, 2014-15 Influenza Season: Centers for Disease Control and Prevention; 2015 [cited 2016 Jan 4]. Available from: <http://www.cdc.gov/flu/fluvaxview/coverage-1415estimates.htm>.

3. Key Facts About Seasonal Flu Vaccine: Centers for Disease Control and Prevention; 2014 [updated October 22, 2014; cited 2015 June 8]. Available from: <http://www.cdc.gov/flu/protect/keyfacts.htm>.

4. Percentage of visits for ILI, National Summary, 2009-10 Season, week ending Oct 02, 2010: Centers for Disease Control and Prevention; 2010 [cited 2016 April 16]. Available from: <http://gis.cdc.gov/grasp/fluview/fluportaldashboard.html>.
