# Supplementary material for: Value of inventory information in allocating a limited supply of influenza vaccine during a pandemic
Source: PLoS One. 2018 Oct 25;13(10):e0206293. doi: 10.1371/journal.pone.0206293 (PMC6201932; doi:10.1371/journal.pone.0206293)
Supplement: S4 Appendix — (DOCX) [file pone.0206293.s004.docx]

In this section we compare the IAR (Tables A to C), inventory leftover (Tables D-F), and vaccine administered (Tables G to I) as a percentage of the total population under PB and PIB across different vaccine distribution horizon (4, 8, and 12 weeks), vaccination start week (week 4 or 7), total supply as a percentage of the total population (20% to 80%), and the uptake rate distributions ($UTR_{1}$, $UTR_{2}$, and $UTR_{3}$). We present the results under PB and PIB for each scenario (as an average of 50 simulation runs).

To compare the results under both strategies, we take the difference between the PB and PIB results and perform statistical tests. All p-values and the corresponding confidence intervals are under two-sample $t$-test with confidence level 0.95. Total vaccine supply is relative to the total population in the state of Georgia in the United States (roughly 10 million).

Tables A to C compare the total attack rate for 3 uptake rate distributions (Table A for $UTR_{1}$, Table B for $UTR_{2}$, and Table C for $UTR_{3}$).

**Table A: Comparison on the total attack rate between PB and PIB for uptake rate distribution** $\boldsymbol{UT}\boldsymbol{R}_{\boldsymbol{1}}$**.**

| **Vaccine distribution horizon (weeks)** | **Start week** | **Total Supply** | **PB** | **PIB** | **Avg percentage points difference PB – PIB** | **95% CI percentage points difference**  **PB - PIB** | | **p-value** |
| --- | --- | --- | --- | --- | --- | --- | --- | --- |
| 4 | 4 | 20% | 27.8% | 27.8% | 0.1 | -0.2 | 0.3 | 0.6121 |
|  |  | 40% | 17$.$0% | 15.4% | 1.6 | 1.3 | 1.9 | 0.0000 |
|  |  | 60% | 12$.$1% | 11.0% | 1.1 | 0.8 | 1.5 | 0.0000 |
|  |  | 80% | 10$.$3% | 10.1% | 0.2 | -0.1 | 0.4 | 0.2978 |
|  | 7 | 20% | 37.8% | 37.9% | -0.1 | -0.5 | 0.2 | 0.4145 |
|  |  | 40% | 31.9% | 31.2% | 0.7 | 0.3 | 1.2 | 0.0026 |
|  |  | 60% | 28.3% | 27.9% | 0.4 | -0.2 | 1.0 | 0.1826 |
|  |  | 80% | 26.0% | 26.2% | -0.2 | -0.8 | 0.4 | 0.5514 |
| 8 | 4 | 20% | 33.5% | 33.5% | -0.1 | -0.4 | 0.2 | 0.7026 |
|  |  | 40% | 23.4% | 22.4% | 1.0 | 0.6 | 1.4 | 0.0000 |
|  |  | 60% | 17.7% | 16.5% | 1.2 | 0.8 | 1.6 | 0.0000 |
|  |  | 80% | 14.5% | 13.9% | 0.7 | 0.2 | 1.1 | 0.0024 |
|  | 7 | 20% | 42.1% | 42.1% | -0.1 | -0.3 | 0.2 | 0.6651 |
|  |  | 40% | 37.1% | 36.7% | 0.3 | 0.0 | 0.7 | 0.0804 |
|  |  | 60% | 33.4% | 33.0% | 0.4 | -0.1 | 0.9 | 0.1348 |
|  |  | 80% | 30.8% | 30.9% | -0.1 | -0.6 | 0.4 | 0.6408 |
| 12 | 4 | 20% | 37.8% | 37.9% | 0.0 | -0.2 | 0.2 | 0.9822 |
|  |  | 40% | 28.8% | 28.4% | 0.4 | 0.0 | 0.8 | 0.0540 |
|  |  | 60% | 22.5% | 22.1% | 0.4 | 0.0 | 0.8 | 0.0663 |
|  |  | 80% | 18.7% | 18.0% | 0.7 | 0.2 | 1.2 | 0.0030 |
|  | 7 | 20% | 44.1% | 44.0% | 0.1 | -0.1 | 0.3 | 0.3899 |
|  |  | 40% | 39.8% | 39.8% | 0.0 | -0.2 | 0.3 | 0.7486 |
|  |  | 60% | 37.0% | 36.8% | 0.1 | -0.3 | 0.6 | 0.4969 |
|  |  | 80% | 34.4% | 34.3% | 0.1 | -0.4 | 0.6 | 0.6855 |

**Table B: Comparison on the total attack rate between PB and PIB for uptake rate distribution** $\boldsymbol{UT}\boldsymbol{R}_{\boldsymbol{2}}$**.**

| **Vaccine distribution horizon (weeks)** | **Start week** | **Total Supply** | **PB** | **PIB** | **Avg percentage points difference PB - PIB** | **95% CI percentage points difference**  **PB - PIB** | | **p-value** |
| --- | --- | --- | --- | --- | --- | --- | --- | --- |
| 4 | 4 | 20% | 39.4% | 33.4% | 6.0 | 5.8 | 6.2 | 0.0000 |
|  |  | 40% | 31.4% | 24.7% | 6.7 | 6.4 | 6.9 | 0.0000 |
|  |  | 60% | 26.1% | 21.4% | 4.7 | 4.4 | 5.0 | 0.0000 |
|  |  | 80% | 22.8% | 20.5% | 2.2 | 1.9 | 2.6 | 0.0000 |
|  | 7 | 20% | 43.8% | 40.9% | 2.9 | 2.7 | 3.2 | 0.0000 |
|  |  | 40% | 39.5% | 36.3% | 3.1 | 2.7 | 3.6 | 0.0000 |
|  |  | 60% | 36.4% | 33.6% | 2.7 | 2.2 | 3.2 | 0.0000 |
|  |  | 80% | 34.2% | 32.6% | 1.6 | 1.1 | 2.0 | 0.0000 |
| 8 | 4 | 20% | 41.9% | 36.8% | 5.1 | 4.9 | 5.3 | 0.0000 |
|  |  | 40% | 35.6% | 29.2% | 6.4 | 6.0 | 6.7 | 0.0000 |
|  |  | 60% | 30.9% | 24.9% | 5.9 | 5.5 | 6.3 | 0.0000 |
|  |  | 80% | 27.2% | 23.1% | 4.0 | 3.7 | 4.4 | 0.0000 |
|  | 7 | 20% | 45.9% | 43.9% | 2.1 | 1.9 | 2.3 | 0.0000 |
|  |  | 40% | 43.0% | 40.0% | 3.0 | 2.7 | 3.2 | 0.0000 |
|  |  | 60% | 40.4% | 37.7% | 2.7 | 2.4 | 3.0 | 0.0000 |
|  |  | 80% | 38.5% | 35.8% | 2.8 | 2.3 | 3.2 | 0.0000 |
| 12 | 4 | 20% | 44.0% | 39.9% | 4.1 | 3.9 | 4.3 | 0.0000 |
|  |  | 40% | 39.2% | 33.3% | 5.8 | 5.6 | 6.1 | 0.0000 |
|  |  | 60% | 34.9% | 28.8% | 6.0 | 5.7 | 6.4 | 0.0000 |
|  |  | 80% | 31.6% | 26.1% | 5.6 | 5.2 | 5.9 | 0.0000 |
|  | 7 | 20% | 47.0% | 45.3% | 1.7 | 1.6 | 1.9 | 0.0000 |
|  |  | 40% | 44.7% | 42.1% | 2.6 | 2.4 | 2.8 | 0.0000 |
|  |  | 60% | 42.6% | 40.1% | 2.5 | 2.2 | 2.8 | 0.0000 |
|  |  | 80% | 40.8% | 38.1% | 2.7 | 2.4 | 3.0 | 0.0000 |

**Table C: Comparison on the total attack rate between PB and PIB for uptake rate distribution** $\boldsymbol{UT}\boldsymbol{R}_{\boldsymbol{3}}$**.**

| **Vaccine distribution horizon (weeks)** | **Start week** | **Total Supply** | **PB** | **PIB** | **Avg percentage points difference PB - PIB** | **95% CI percentage points difference**  **PB - PIB** | | **p-value** |
| --- | --- | --- | --- | --- | --- | --- | --- | --- |
| 4 | 4 | 20% | 29.9% | 28.7% | 1.2 | 1.0 | 1.4 | 0.0000 |
|  |  | 40% | 18.5% | 16.5% | 2.0 | 1.6 | 2.3 | 0.0000 |
|  |  | 60% | 13.8% | 12.7% | 1.1 | 0.8 | 1.4 | 0.0000 |
|  |  | 80% | 11.9% | 11.5% | 0.4 | 0.1 | 0.8 | 0.0230 |
|  | 7 | 20% | 38.8% | 38.3% | 0.4 | 0.1 | 0.8 | 0.0223 |
|  |  | 40% | 32.5% | 31.7% | 0.8 | 0.2 | 1.3 | 0.0054 |
|  |  | 60% | 29.5% | 28.4% | 1.1 | 0.6 | 1.6 | 0.0001 |
|  |  | 80% | 27.5% | 26.7% | 0.7 | 0.2 | 1.3 | 0.0098 |
| 8 | 4 | 20% | 34.8% | 33.9% | 0.9 | 0.6 | 1.2 | 0.0000 |
|  |  | 40% | 25.0% | 23.4% | 1.6 | 1.2 | 2.0 | 0.0000 |
|  |  | 60% | 19.2% | 17.5% | 1.6 | 1.2 | 2.0 | 0.0000 |
|  |  | 80% | 16.2% | 15.1% | 1.1 | 0.7 | 1.4 | 0.0000 |
|  | 7 | 20% | 42.5% | 42.3% | 0.2 | -0.1 | 0.4 | 0.1828 |
|  |  | 40% | 37.9% | 37.1% | 0.8 | 0.5 | 1.2 | 0.0000 |
|  |  | 60% | 34.6% | 33.6% | 1.0 | 0.6 | 1.5 | 0.0000 |
|  |  | 80% | 32.0% | 31.7% | 0.3 | -0.2 | 0.8 | 0.2278 |
| 12 | 4 | 20% | 38.7% | 38.1% | 0.6 | 0.4 | 0.8 | 0.0000 |
|  |  | 40% | 30.4% | 29.0% | 1.4 | 1.0 | 1.8 | 0.0000 |
|  |  | 60% | 24.7% | 22.8% | 1.9 | 1.4 | 2.3 | 0.0000 |
|  |  | 80% | 20.5% | 18.8% | 1.7 | 1.2 | 2.3 | 0.0000 |
|  | 7 | 20% | 44.4% | 44.4% | 0.1 | -0.1 | 0.3 | 0.5262 |
|  |  | 40% | 40.4% | 40.0% | 0.4 | 0.1 | 0.6 | 0.0171 |
|  |  | 60% | 37.8% | 37.0% | 0.8 | 0.4 | 1.2 | 0.0001 |
|  |  | 80% | 35.0% | 34.4% | 0.6 | 0.1 | 1.0 | 0.0126 |

Tables D to F compare the total inventory leftover as a percentage of the total population for 3 uptake rate distributions (Table D for $UTR_{1}$, Table E for $UTR_{2}$ , and Table F for $UTR_{3}$).

**Table D: Comparison on the total inventory leftover as a percentage of the total population between PB and PIB for uptake rate distribution** $\boldsymbol{UT}\boldsymbol{R}_{\boldsymbol{1}}$**.**

| **Vaccine distribution horizon (weeks)** | **Start week** | **Total Supply** | **PB** | **PIB** | **Avg percentage points difference PB - PIB** | **95% CI percentage points difference**  **PB - PIB** | | **p-value** |
| --- | --- | --- | --- | --- | --- | --- | --- | --- |
| 4 | 4 | 20% | 0.0% | 0.0% | 0.0 | 0.0 | 0.0 | 0.0006 |
|  |  | 40% | 7.9% | 2.9% | 5.0 | 4.9 | 5.0 | 0.0000 |
|  |  | 60% | 17.7% | 11.5% | 6.3 | 6.1 | 6.4 | 0.0000 |
|  |  | 80% | 31.5% | 11.3% | 20.2 | 20.1 | 20.4 | 0.0000 |
|  | 7 | 20% | 0.1% | 0.1% | 0.0 | 0.0 | 0.0 | 0.9814 |
|  |  | 40% | 8.5% | 3.4% | 5.1 | 5.0 | 5.2 | 0.0000 |
|  |  | 60% | 18.5% | 15.1% | 3.4 | 3.2 | 3.6 | 0.0000 |
|  |  | 80% | 34.6% | 14.3% | 20.3 | 20.1 | 20.5 | 0.0000 |
| 8 | 4 | 20% | 0.1% | 0.1% | 0.0 | 0.0 | 0.0 | 0.0000 |
|  |  | 40% | 8.3% | 1.5% | 6.7 | 6.7 | 6.8 | 0.0000 |
|  |  | 60% | 18.0% | 6.0% | 12.1 | 11.9 | 12.2 | 0.0000 |
|  |  | 80% | 33.1% | 11.8% | 21.3 | 21.1 | 21.6 | 0.0000 |
|  | 7 | 20% | 1.0% | 0.7% | 0.3 | 0.3 | 0.4 | 0.0000 |
|  |  | 40% | 9.4% | 3.3% | 6.1 | 6.0 | 6.3 | 0.0000 |
|  |  | 60% | 20.8% | 8.9% | 11.9 | 11.7 | 12.2 | 0.0000 |
|  |  | 80% | 38.4% | 8.3% | 30.0 | 29.8 | 30.2 | 0.0000 |
| 12 | 4 | 20% | 0.7% | 0.4% | 0.3 | 0.2 | 0.3 | 0.0000 |
|  |  | 40% | 8.8% | 1.5% | 7.3 | 7.2 | 7.4 | 0.0000 |
|  |  | 60% | 18.7% | 5.9% | 12.8 | 12.6 | 13.0 | 0.0000 |
|  |  | 80% | 35.2% | 7.3% | 27.9 | 27.7 | 28.1 | 0.0000 |
|  | 7 | 20% | 1.9% | 0.6% | 1.3 | 1.3 | 1.3 | 0.0000 |
|  |  | 40% | 10.4% | 5.4% | 4.9 | 4.8 | 5.1 | 0.0000 |
|  |  | 60% | 23.8% | 7.4% | 16.4 | 16.2 | 16.6 | 0.0000 |
|  |  | 80% | 41.5% | 7.3% | 34.2 | 34.0 | 34.4 | 0.0000 |

**Table E: Comparison on the total inventory leftover as a percentage of the total population between PB and PIB for uptake rate distribution** $\boldsymbol{UT}\boldsymbol{R}_{\boldsymbol{2}}$**.**

| **Vaccine distribution horizon (weeks)** | **Start week** | **Total Supply** | **PB** | **PIB** | **Avg percentage points difference PB - PIB** | **95% CI percentage points difference**  **PB - PIB** | | **p-value** |
| --- | --- | --- | --- | --- | --- | --- | --- | --- |
| 4 | 4 | 20% | 2.2% | 0.7% | 1.5 | 1.5 | 1.5 | 0.0000 |
|  |  | 40% | 8.4% | 3.4% | 5.0 | 4.9 | 5.1 | 0.0000 |
|  |  | 60% | 18.7% | 12.0% | 6.7 | 6.5 | 6.9 | 0.0000 |
|  |  | 80% | 33.2% | 13.8% | 19.4 | 19.2 | 19.6 | 0.0000 |
|  | 7 | 20% | 2.3% | 0.8% | 1.6 | 1.5 | 1.6 | 0.0000 |
|  |  | 40% | 9.0% | 4.0% | 5.0 | 4.9 | 5.1 | 0.0000 |
|  |  | 60% | 20.1% | 15.3% | 4.9 | 4.7 | 5.1 | 0.0000 |
|  |  | 80% | 35.6% | 15.2% | 20.4 | 20.2 | 20.6 | 0.0000 |
| 8 | 4 | 20% | 2.3% | 0.4% | 1.9 | 1.9 | 1.9 | 0.0000 |
|  |  | 40% | 8.8% | 2.2% | 6.5 | 6.4 | 6.6 | 0.0000 |
|  |  | 60% | 19.5% | 7.4% | 12.0 | 11.9 | 12.2 | 0.0000 |
|  |  | 80% | 34.3% | 9.9% | 24.4 | 24.2 | 24.6 | 0.0000 |
|  | 7 | 20% | 2.7% | 0.5% | 2.1 | 2.1 | 2.2 | 0.0000 |
|  |  | 40% | 10.2% | 3.9% | 6.3 | 6.2 | 6.5 | 0.0000 |
|  |  | 60% | 22.6% | 9.1% | 13.5 | 13.3 | 13.7 | 0.0000 |
|  |  | 80% | 39.1% | 9.6% | 29.4 | 29.2 | 29.7 | 0.0000 |
| 12 | 4 | 20% | 2.6% | 0.3% | 2.2 | 2.2 | 2.3 | 0.0000 |
|  |  | 40% | 9.5% | 2.2% | 7.3 | 7.2 | 7.4 | 0.0000 |
|  |  | 60% | 20.8% | 6.3% | 14.5 | 14.3 | 14.7 | 0.0000 |
|  |  | 80% | 36.1% | 7.6% | 28.5 | 28.2 | 28.7 | 0.0000 |
|  | 7 | 20% | 3.1% | 0.5% | 2.6 | 2.6 | 2.6 | 0.0000 |
|  |  | 40% | 11.4% | 5.5% | 5.9 | 5.7 | 6.0 | 0.0000 |
|  |  | 60% | 25.0% | 7.5% | 17.5 | 17.3 | 17.7 | 0.0000 |
|  |  | 80% | 42.0% | 7.8% | 34.3 | 34.0 | 34.5 | 0.0000 |

**Table F: Comparison on the total inventory leftover as a percentage of the total population between PB and PIB for uptake rate distribution** $\boldsymbol{UT}\boldsymbol{R}_{\boldsymbol{3}}$**.**

| **Vaccine distribution horizon (weeks)** | **Start week** | **Total Supply** | **PB** | **PIB** | **Avg percentage points difference PB - PIB** | **95% CI percentage points difference**  **PB - PIB** | | **p-value** |
| --- | --- | --- | --- | --- | --- | --- | --- | --- |
| 4 | 4 | 20% | 10.2% | 2.5% | 7.6 | 7.5 | 7.7 | 0.0000 |
|  |  | 40% | 20.1% | 5.0% | 15.1 | 14.9 | 15.3 | 0.0000 |
|  |  | 60% | 30.1% | 8.3% | 21.8 | 21.6 | 22.1 | 0.0000 |
|  |  | 80% | 40.1% | 10.5% | 29.6 | 29.3 | 30.0 | 0.0000 |
|  | 7 | 20% | 10.2% | 2.5% | 7.6 | 7.5 | 7.7 | 0.0000 |
|  |  | 40% | 20.1% | 5.1% | 15.0 | 14.9 | 15.2 | 0.0000 |
|  |  | 60% | 30.1% | 12.2% | 18.0 | 17.7 | 18.2 | 0.0000 |
|  |  | 80% | 40.4% | 13.9% | 26.5 | 26.1 | 26.8 | 0.0000 |
| 8 | 4 | 20% | 10.3% | 1.3% | 9.0 | 9.0 | 9.1 | 0.0000 |
|  |  | 40% | 20.3% | 2.6% | 17.7 | 17.6 | 17.9 | 0.0000 |
|  |  | 60% | 30.3% | 5.7% | 24.6 | 24.3 | 24.9 | 0.0000 |
|  |  | 80% | 40.3% | 6.3% | 34.0 | 33.7 | 34.3 | 0.0000 |
|  | 7 | 20% | 10.3% | 1.3% | 9.0 | 9.0 | 9.1 | 0.0000 |
|  |  | 40% | 20.3% | 4.2% | 16.1 | 15.9 | 16.3 | 0.0000 |
|  |  | 60% | 30.3% | 7.6% | 22.7 | 22.5 | 23.0 | 0.0000 |
|  |  | 80% | 43.0% | 8.8% | 34.2 | 33.8 | 34.6 | 0.0000 |
| 12 | 4 | 20% | 10.5% | 0.9% | 9.6 | 9.5 | 9.7 | 0.0000 |
|  |  | 40% | 20.5% | 2.1% | 18.4 | 18.3 | 18.6 | 0.0000 |
|  |  | 60% | 30.5% | 5.2% | 25.2 | 25.0 | 25.5 | 0.0000 |
|  |  | 80% | 41.0% | 5.7% | 35.3 | 34.9 | 35.6 | 0.0000 |
|  | 7 | 20% | 10.5% | 0.9% | 9.6 | 9.5 | 9.7 | 0.0000 |
|  |  | 40% | 20.5% | 5.2% | 15.3 | 15.1 | 15.4 | 0.0000 |
|  |  | 60% | 30.8% | 6.7% | 24.1 | 23.9 | 24.4 | 0.0000 |
|  |  | 80% | 45.8% | 7.9% | 37.9 | 37.6 | 38.3 | 0.0000 |

Tables G to I compare the total vaccine administered as a percentage of the total population for 3 uptake rate distributions (Table G for $UTR_{1}$, Table H for $UTR_{2}$, and Table I for $UTR_{3}$). Note that PIB administer more vaccine than PB, so we use PIB - PB instead of PB - PIB.

**Table G: Comparison on the total vaccine administered as a percentage of the total population between PB and PIB for uptake rate distribution** $\boldsymbol{UT}\boldsymbol{R}_{\boldsymbol{1}}$**.**

| **Vaccine distribution horizon (weeks)** | **Start week** | **Total Supply** | **PB** | **PIB** | **Avg percentage points difference PIB - PB** | **95% CI percentage points difference PIB - PB** | | **p-value** |
| --- | --- | --- | --- | --- | --- | --- | --- | --- |
| 4 | 4 | 20% | 20.0% | 20.0% | 0.0 | 0.0 | 0.0 | 0.0006 |
|  |  | 40% | 32.1% | 37.1% | 5.0 | 4.9 | 5.0 | 0.0000 |
|  |  | 60% | 42.3% | 48.5% | 6.3 | 6.1 | 6.4 | 0.0000 |
|  |  | 80% | 48.5% | 48.7% | 0.2 | 0.1 | 0.4 | 0.0117 |
|  | 7 | 20% | 19.9% | 19.9% | 0.0 | 0.0 | 0.0 | 0.9814 |
|  |  | 40% | 31.5% | 36.6% | 5.1 | 5.0 | 5.2 | 0.0000 |
|  |  | 60% | 41.5% | 44.9% | 3.4 | 3.2 | 3.6 | 0.0000 |
|  |  | 80% | 45.4% | 45.7% | 0.3 | 0.1 | 0.5 | 0.0164 |
| 8 | 4 | 20% | 19.9% | 19.9% | 0.0 | 0.0 | 0.0 | 0.0000 |
|  |  | 40% | 31.7% | 38.5% | 6.7 | 6.7 | 6.8 | 0.0000 |
|  |  | 60% | 42.0% | 46.7% | 4.8 | 4.6 | 4.9 | 0.0000 |
|  |  | 80% | 46.9% | 47.7% | 0.9 | 0.7 | 1.0 | 0.0000 |
|  | 7 | 20% | 19.0% | 19.3% | 0.3 | 0.3 | 0.4 | 0.0000 |
|  |  | 40% | 30.6% | 36.7% | 6.1 | 6.0 | 6.3 | 0.0000 |
|  |  | 60% | 39.2% | 40.8% | 1.6 | 1.4 | 1.8 | 0.0000 |
|  |  | 80% | 41.6% | 42.7% | 1.0 | 0.8 | 1.3 | 0.0000 |
| 12 | 4 | 20% | 19.3% | 19.6% | 0.3 | 0.2 | 0.3 | 0.0000 |
|  |  | 40% | 31.2% | 38.5% | 7.3 | 7.2 | 7.4 | 0.0000 |
|  |  | 60% | 41.3% | 44.1% | 2.8 | 2.6 | 3.0 | 0.0000 |
|  |  | 80% | 44.8% | 46.2% | 1.4 | 1.2 | 1.5 | 0.0000 |
|  | 7 | 20% | 18.1% | 19.4% | 1.3 | 1.3 | 1.3 | 0.0000 |
|  |  | 40% | 29.6% | 34.2% | 4.5 | 4.4 | 4.7 | 0.0000 |
|  |  | 60% | 36.2% | 37.3% | 1.1 | 0.8 | 1.3 | 0.0000 |
|  |  | 80% | 38.5% | 39.7% | 1.3 | 1.0 | 1.5 | 0.0000 |

**Table H: Comparison on the total vaccine administered as a percentage of the total population between PB and PIB for uptake rate distribution** $\boldsymbol{UT}\boldsymbol{R}_{\boldsymbol{2}}$**.**

| **Vaccine distribution horizon (weeks)** | **Start week** | **Total Supply** | **PB** | **PIB** | **Avg percentage points difference PIB - PB** | **95% CI PIB - PB** | | **p-value** |
| --- | --- | --- | --- | --- | --- | --- | --- | --- |
| 4 | 4 | 20% | 17.8% | 19.3% | 1.5 | 1.5 | 1.5 | 0.0000 |
|  |  | 40% | 31.6% | 36.6% | 5.0 | 4.9 | 5.1 | 0.0000 |
|  |  | 60% | 41.3% | 48.0% | 6.7 | 6.5 | 6.9 | 0.0000 |
|  |  | 80% | 46.8% | 48.5% | 1.6 | 1.4 | 1.9 | 0.0000 |
|  | 7 | 20% | 17.7% | 19.2% | 1.6 | 1.5 | 1.6 | 0.0000 |
|  |  | 40% | 31.0% | 36.0% | 5.0 | 4.9 | 5.1 | 0.0000 |
|  |  | 60% | 39.9% | 44.6% | 4.7 | 4.5 | 5.0 | 0.0000 |
|  |  | 80% | 44.4% | 45.6% | 1.2 | 0.9 | 1.5 | 0.0000 |
| 8 | 4 | 20% | 17.7% | 19.6% | 1.9 | 1.9 | 1.9 | 0.0000 |
|  |  | 40% | 31.2% | 37.8% | 6.5 | 6.4 | 6.6 | 0.0000 |
|  |  | 60% | 40.5% | 46.5% | 5.9 | 5.7 | 6.2 | 0.0000 |
|  |  | 80% | 45.7% | 47.4% | 1.7 | 1.5 | 2.0 | 0.0000 |
|  | 7 | 20% | 17.3% | 19.5% | 2.1 | 2.1 | 2.2 | 0.0000 |
|  |  | 40% | 29.8% | 36.1% | 6.3 | 6.2 | 6.5 | 0.0000 |
|  |  | 60% | 37.4% | 40.5% | 3.1 | 2.8 | 3.3 | 0.0000 |
|  |  | 80% | 40.9% | 42.3% | 1.4 | 1.1 | 1.7 | 0.0000 |
| 12 | 4 | 20% | 17.4% | 19.7% | 2.2 | 2.2 | 2.3 | 0.0000 |
|  |  | 40% | 30.5% | 37.8% | 7.3 | 7.2 | 7.4 | 0.0000 |
|  |  | 60% | 39.2% | 43.9% | 4.7 | 4.4 | 5.0 | 0.0000 |
|  |  | 80% | 43.9% | 45.9% | 2.0 | 1.7 | 2.3 | 0.0000 |
|  | 7 | 20% | 16.9% | 19.5% | 2.6 | 2.6 | 2.6 | 0.0000 |
|  |  | 40% | 28.6% | 34.0% | 5.3 | 5.2 | 5.5 | 0.0000 |
|  |  | 60% | 35.0% | 37.2% | 2.2 | 2.0 | 2.5 | 0.0000 |
|  |  | 80% | 38.0% | 39.6% | 1.7 | 1.3 | 2.0 | 0.0000 |

**Table I: Comparison on the total vaccine administered as a percentage of the total population between PB and PIB for uptake rate distribution** $\boldsymbol{UT}\boldsymbol{R}_{\boldsymbol{3}}$**.**

| **Vaccine distribution horizon (weeks)** | **Start week** | **Total Supply** | **PB** | **PIB** | **Avg percentage points difference PIB - PB** | **95% CI percentage points difference**  **PIB - PB** | | **p-value** |
| --- | --- | --- | --- | --- | --- | --- | --- | --- |
| 4 | 4 | 20% | 9.8% | 17.5% | 7.6 | 7.5 | 7.7 | 0.0000 |
|  |  | 40% | 19.9% | 35.0% | 15.1 | 14.9 | 15.3 | 0.0000 |
|  |  | 60% | 29.9% | 48.2% | 18.4 | 17.9 | 18.8 | 0.0000 |
|  |  | 80% | 39.9% | 48.6% | 8.7 | 8.2 | 9.2 | 0.0000 |
|  | 7 | 20% | 9.8% | 17.5% | 7.6 | 7.5 | 7.7 | 0.0000 |
|  |  | 40% | 19.9% | 34.9% | 15.0 | 14.9 | 15.2 | 0.0000 |
|  |  | 60% | 29.9% | 44.1% | 14.2 | 13.7 | 14.7 | 0.0000 |
|  |  | 80% | 39.6% | 45.0% | 5.4 | 4.9 | 5.9 | 0.0000 |
| 8 | 4 | 20% | 9.7% | 18.7% | 9.0 | 9.0 | 9.1 | 0.0000 |
|  |  | 40% | 19.7% | 37.4% | 17.7 | 17.6 | 17.9 | 0.0000 |
|  |  | 60% | 29.7% | 46.5% | 16.8 | 16.4 | 17.2 | 0.0000 |
|  |  | 80% | 39.7% | 47.4% | 7.8 | 7.3 | 8.2 | 0.0000 |
|  | 7 | 20% | 9.7% | 18.7% | 9.0 | 9.0 | 9.1 | 0.0000 |
|  |  | 40% | 19.7% | 35.8% | 16.1 | 15.9 | 16.3 | 0.0000 |
|  |  | 60% | 29.7% | 39.8% | 10.2 | 9.8 | 10.6 | 0.0000 |
|  |  | 80% | 37.0% | 42.0% | 5.0 | 4.5 | 5.5 | 0.0000 |
| 12 | 4 | 20% | 9.5% | 19.1% | 9.6 | 9.5 | 9.7 | 0.0000 |
|  |  | 40% | 19.5% | 37.9% | 18.4 | 18.3 | 18.6 | 0.0000 |
|  |  | 60% | 29.5% | 43.9% | 14.4 | 14.0 | 14.8 | 0.0000 |
|  |  | 80% | 39.0% | 45.8% | 6.8 | 6.4 | 7.3 | 0.0000 |
|  | 7 | 20% | 9.5% | 19.1% | 9.6 | 9.5 | 9.7 | 0.0000 |
|  |  | 40% | 19.5% | 33.8% | 14.3 | 14.0 | 14.6 | 0.0000 |
|  |  | 60% | 29.2% | 36.6% | 7.4 | 7.1 | 7.8 | 0.0000 |
|  |  | 80% | 34.2% | 39.1% | 5.0 | 4.5 | 5.4 | 0.0000 |
