# Supplementary material for: Value of inventory information in allocating a limited supply of influenza vaccine during a pandemic
Source: PLoS One. 2018 Oct 25;13(10):e0206293. doi: 10.1371/journal.pone.0206293 (PMC6201932; doi:10.1371/journal.pone.0206293)
Supplement: S5 Appendix — (DOCX) [file pone.0206293.s005.docx]

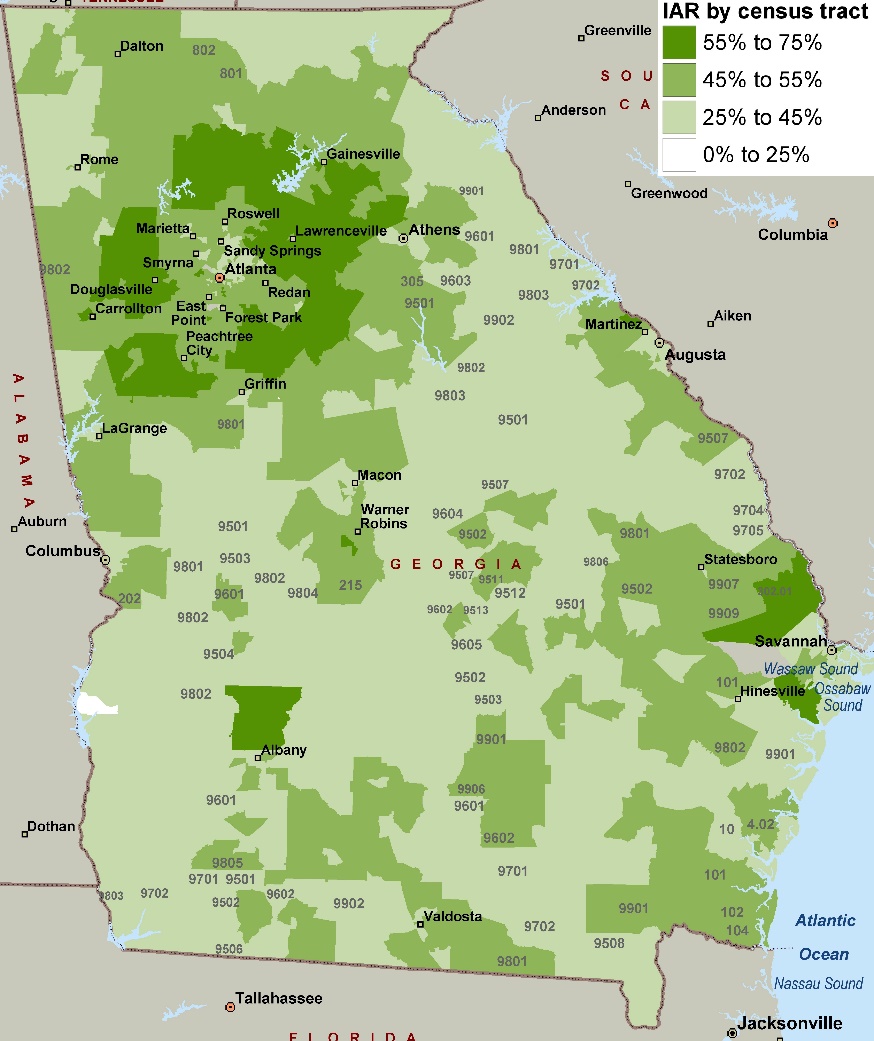


**Fig A: Total infection attack rate (IAR) of each census tract in the state of Georgia when vaccination is not available.**

Prior studies suggest that the overall impact of vaccination can be sensitive to the start time of vaccination and the amount of vaccine available [[1](#_ENREF_1)].

Fig B shows the prevalence (percentage of population infected) under no vaccination and vaccination following the PB strategy when the total vaccine supply is equal to 40% of the total population.

**Fig B: Disease prevalence in Georgia under (i) no vaccination and (ii) PB strategy when the vaccination start week is week 4 or 7, and the vaccine distribution horizon is 4 weeks or 8 weeks.**

Fig C compares the IAR when vaccination is not available and when vaccination is available for different vaccine supply levels under PB. Fig C1 shows the comparison of IAR when the vaccination start week is 4 or 7, and the vaccine distribution horizon is 4 weeks; Fig C2 is similar but the vaccine distribution horizon is 8 weeks. IAR is lower for each scenario in Fig C1 compared to C2. Starting vaccination in week 4 instead of week 7 reduces the IAR from 37.1% to 23.4% under PB when the vaccine supply is sufficient to cover 40% of population and the vaccine distribution horizon is 8 weeks.

The importance of the vaccination start week can even outweigh the importance of the total vaccine supply. For example, consider the two scenarios where (i) vaccine supply is equivalent to 20% of the population, vaccine distribution horizon is 4 weeks, and vaccine start week is week 4 (IAR = 27.8% for PB), and (ii) vaccine supply is 80% of the population, vaccine distribution horizon is 8 weeks, and vaccine start week is week 7 (IAR = 30.8% for PB). Even though the vaccine inventory is four times higher under (ii) versus (i), IAR is higher under (ii) because most of the vaccine arrives after the “peak”. These observations about the importance of the vaccination start week are consistent with the previous findings in the literature [[2-4](#_ENREF_2)].

**Fig C: IAR comparison under different vaccine supply levels when the vaccination start week is 4 or 7. C1: vaccine distribution horizon is 4 weeks. C2: vaccine distribution horizon is 8 weeks.**

**References:**

1. Feng Z, Towers S, Yang Y. Modeling the effects of vaccination and treatment on pandemic influenza. AAPS J. 2011;13(3):427-37. doi: 10.1208/s12248-011-9284-7. PubMed PMID: 21656080; PubMed Central PMCID: PMCPMC3160165.

2. Davila-Payan C, DeGuzman M, Johnson K, Serban N, Swann J. Estimating Prevalence of Overweight or Obese Children and Adolescents in Small Geographic Areas Using Publicly Available Data. Preventing Chronic Disease. 2015;12. doi: 10.5888/pcd12.140229. PubMed PMID: WOS:000356669000005.

3. Davila-Payan C, Swann J, Wortley PM. System factors to explain 2009 pandemic H1N1 state vaccination rates for children and high-risk adults in US emergency response to pandemic. Vaccine. 2014;32(2):246-51. doi: 10.1016/j.vaccine.2013.11.018. PubMed PMID: WOS:000329772900009.

4. Ekici A, Keskinocak P, Swann JL. Modeling Influenza Pandemic and Planning Food Distribution. M&Som-Manufacturing & Service Operations Management. 2014;16(1):11-27. doi: 10.1287/msom.2013.0460. PubMed PMID: WOS:000343747400002.
