# Supplementary material for: Value of inventory information in allocating a limited supply of influenza vaccine during a pandemic
Source: PLoS One. 2018 Oct 25;13(10):e0206293. doi: 10.1371/journal.pone.0206293 (PMC6201932; doi:10.1371/journal.pone.0206293)
Supplement: S6 Appendix — (DOCX) [file pone.0206293.s006.docx]

**Additional analysis and discussion on PB vs. PIB**

## **Network effect**

Fig 9 shows the network effects when the uptake rates are correlated with education levels (census tracts with 82.4% population (age $\geq$ 18) with high school or higher degrees have uptake 75%, others have 25%). The IAR under PIB is 0.2 (95% CI, 0.1 to 0.3, $p=$ 0$.$0017) percentage points less than that under PB on average for census tracts with 25% uptake rate and 0.93 (0.8 to 1.0, $p<$ 0$.$0001) percentage points less for census tracts with 75% uptake rate.

## **Herd Immunity**

Interestingly, when vaccine supply is low (equivalent to 20% of the population, vaccine distribution horizon is four weeks and vaccine start week is four in Figure 6), the reduction in IAR is equivalent to 22.5% of the population (from 50.5% to 28.0%), which is more than the vaccine supplied. However, when vaccine supply is high (equivalent to 80% of the population), the incremental impact of an additional vaccination is less (IAR reduces from 50.5% down to 8.0%). This is in part because some of the vaccines are unused since the average uptake rate is 50.5%.


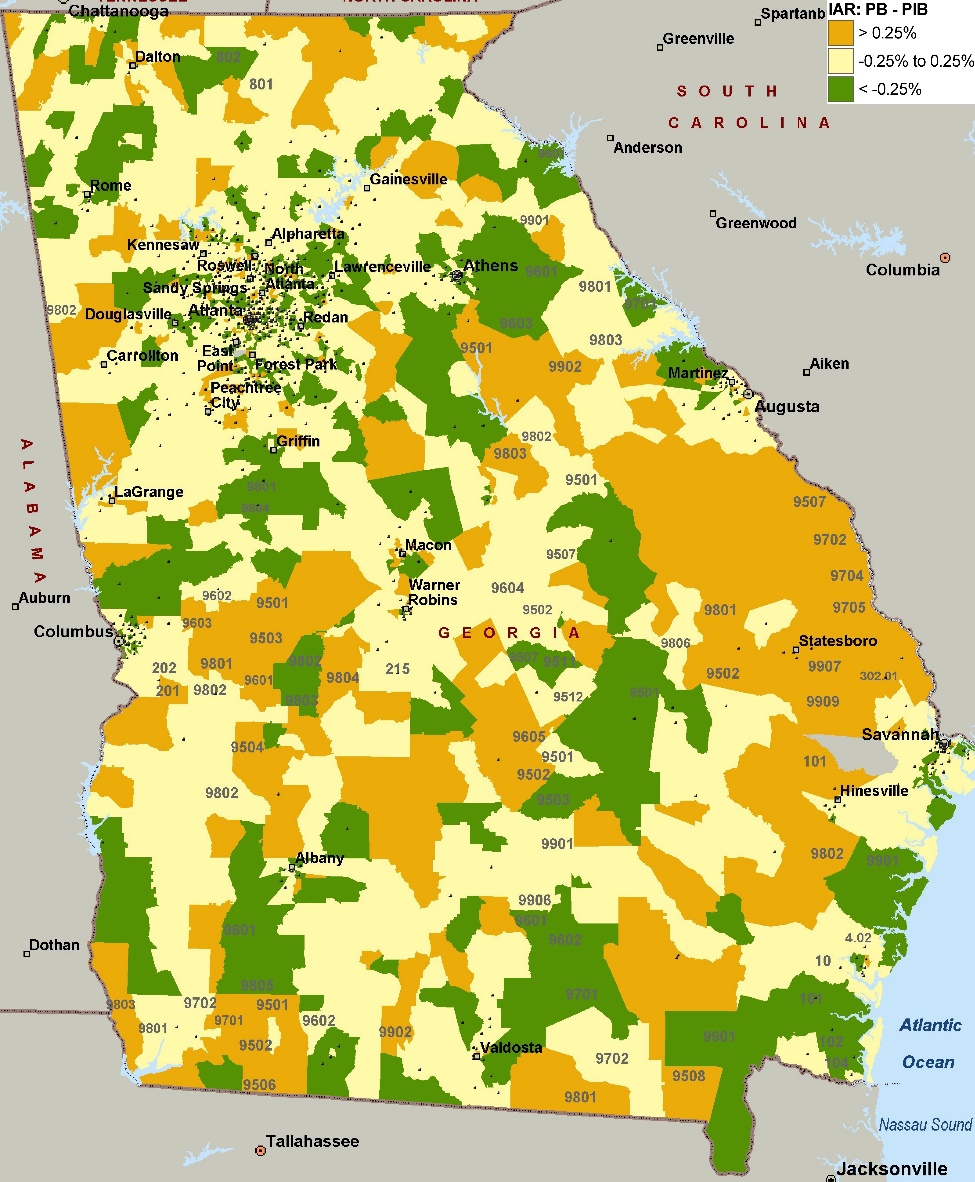


**Fig 9: IAR difference between PIB and PB at county level. PIB has higher IAR in green counties and lower IAR in orange counties. Dots are census tracts with 75% uptake rate.**
